# Parental emotional support, self-efficacy, and mental health problems among adolescents in Hong Kong: a moderated mediation approach

**DOI:** 10.3389/fpsyt.2024.1458275

**Published:** 2024-10-14

**Authors:** Mengting Qian, Rui Jin, Chunping Lu, Mingren Zhao

**Affiliations:** ^1^ School of Psychology, Shenzhen University, Shenzhen, Guangdong, China; ^2^ Faculty of Education, Shenzhen University, Shenzhen, Guangdong, China

**Keywords:** parental emotional support, self-efficacy, mental health problems, adolescent, Hong Kong

## Abstract

**Introduction:**

Early signs of mental health issues may develop into severe mental illnesses over time. The influence of parental emotional support on adolescent mental health problems is well acknowledged. However, prior research has predominantly focused on psychological symptoms, often neglecting the somatic symptoms associated with mental health. Additionally, there has been insufficient exploration of the mediating role of self-efficacy and the relationship between socioeconomic status (SES) and adolescent mental health, particularly within the context of Hong Kong.

**Methods:**

Using data from 3,613 adolescents aged 15 years from 109 schools in Hong Kong who participated in the Programme for International Student Assessment 2018, this study explored the relationship between parental emotional support and two dimensions of adolescent mental health symptoms (psychological and somatic symptoms), and the mediating effect of self-efficacy and the moderating role of SES.

**Results:**

We found that parental emotional support significantly reduced psychological and somatic symptoms, primarily by enhancing self-efficacy. MoreovSES moderated these relationships, with the impact of parental emotional support on psychological symptoms being more pronounced among adolescents from lower SES backgrounds.

**Discussion:**

This study deepens the understanding of the mechanisms underlying adolescent mental health in Hong Kong. By highlighting the importance of parental emotional support and self-efficacy, as well as the moderating effect of SES, the findings offer valuable insights for effective interventions aimed at improving adolescent mental health.

## 1 Introduction

Adolescence is a crucial developmental period during which individuals are susceptible to mental health problems (1). The long-term consequences of these problems can extend into adulthood, particularly when behavioral patterns become entrenched during adolescence (2). Optimal mental health status plays a vital role in facilitating the successful integration of adolescents into society (3, 4). However, mental health problems among adolescents remain largely unrecognized and untreated, affecting approximately 14% of adolescents globally (5). Early non-clinical psychological symptoms, such as anxiety and depression, serve as indicators of mental health problems. If these symptoms go unnoticed and unresolved, they can cause irreversible psychological damage to adolescents, impose financial burdens on families, and, in severe cases, impact societal stability (6, 7). Therefore, early intervention is crucial to prevent the worsening of these psychological symptoms.

Numerous researchers have sought to elucidate the mechanisms by which family support influences adolescent mental health from an ecosystem perspective (8, 9). For instance, empirical studies have demonstrated that effective family support can reduce the risk of mental health issues (10, 11). Specifically, family support provides emotional warmth and understanding, which alleviates feelings of loneliness and stress among adolescents. This positive interaction promotes the development of adaptive emotional regulation skills, enabling adolescents to regulate negative emotions more effectively (12). Furthermore, family support can cultivate adolescents’ psychological functioning (13). For example, as a protective factor, psychological functioning (e.g., self-efficacy) enables adolescents to maintain social function (14). Well-developed social function contributes to establishing and maintaining positive social relationships, reducing negative feelings, and thereby enhancing overall health and well-being (15).

Although these studies provide a substantial theoretical foundation for early intervention in adolescent mental health problems, several significant gaps remain in the literature. First, a great deal of research has only focused on one facet of mental health, primarily psychological symptoms (16). Mental health, however, is a multi-faceted construct consisting of psychological and somatic components (17). Understanding these dimensions collectively is crucial for a comprehensive assessment of adolescent mental health. By including both psychological and somatic symptoms, this study will provide a more holistic perspective on adolescent mental health, which is essential for developing effective interventions.

Second, not all individuals lacking parental emotional support develop mental health problems, indicating the presence of protective factors. Social cognitive theory and previous research have suggested that self-efficacy is a potential mediating variable (18), but this hypothesis has not been empirically tested in adolescents from Hong Kong. Testing this hypothesis in the unique sociocultural context of Hong Kong is critical, as cultural variations may influence the role of self-efficacy in buffering against mental health issues. This study aims to fill this gap by examining whether self-efficacy mediates the relationship between parental emotional support and mental health outcomes among Hong Kong adolescents.

Finally, the third critical gap pertains to the mixed findings regarding the relationship between socioeconomic status (SES) and adolescent mental health. Some studies have found that adolescents with higher SES are at greater risk of mental health problems (19). Conversely, other empirical studies show that adolescents from disadvantaged backgrounds may experience an amplified negative impact of the social environment on their mental health (20). Given that adolescence is an important stage in the transition from childhood dependence on family SES to adulthood, this study explores how SES moderates the relationship between parental emotional support and mental health, thereby clarifying the specific impact of SES during this developmental period in the Hong Kong context.

To address these critical gaps, we used secondary data from the Programme for International Student Assessment (PISA) 2018 and examined how parental emotional support influences adolescents’ mental health (i.e., psychological symptoms and somatic symptoms) via self-efficacy and moderated by SES in the context of Hong Kong.

## 2 Literature review

### 2.1 Two dimensions of mental health problems for adolescents

Mental health typically indicates an individual’s psychological state, manifested in non-clinical psychological and somatic symptoms (21). These symptoms can exert a profound impact on the lives of adolescents (6, 7). For instance, the psychological distress resulting from mental health problems may lead to heightened stress levels, which, in turn, can contribute to maladaptive coping mechanisms, including self-harm or suicidal ideation (22). Similarly, the somatic symptoms associated with mental health problems can affect concentration and engagement in academic activities, potentially leading to a decline in academic performance and an increased likelihood of dropping out (23, 24). These problems can predict social development in adulthood (25).

While previous research has frequently concentrated on psychological facets such as anxiety and depression symptoms (26, 27), the somatic dimension of mental health has often been overlooked. However, non-clinical somatic symptoms, such as headaches or backaches, are integral components of an individual’s mental health status and are deeply intertwined with psychological symptoms (28, 29). A comprehensive understanding of mental health must include both dimensions to ensure accurate assessment and effective intervention strategies. This study addresses this gap by including both psychological and somatic symptoms in its analysis, thereby contributing to a more nuanced understanding of adolescent mental health (30).

Furthermore, somatic symptoms may even obscure psychological symptoms, thereby impacting assessment accuracy (7, 31). From one perspective, emotional events can stimulate the neuroendocrine system and affect physiological manifestations, such as sweating and rapid heartbeat, thus masking psychological symptoms (32). On the other hand, somatic and psychological symptoms may be related to social structure (33). Loose social structure emphasizes the expression of direct emotions, while highly tight social structure encourages individuals to avoid the expression of negative emotions; in the latter case, individuals are more inclined to express feelings indirectly through the presentation of physical symptoms. Therefore, psychological and somatic symptoms are intertwined and constitute two aspects of mental health. An in-depth study of this interlinkage is particularly important in the context of enabling comprehensive mental health assessments.

Empirically, PISA 2018 assessed psychological complaints involving reports of non-clinical psychological distress, such as nervousness or irritability, and somatic complaints, consisting of reports of non-clinical somatic discomfort, such as headaches or backaches.^1^ Please refer to our Methods section for a more in-depth understanding of the “Mental Health” segment.

### 2.2 Parental emotional support, self-efficacy, and mental health

Research has revealed that social support plays a significant and powerful role in maintaining mental health (10, 11, 34). According to attachment theory (35), parental emotional support is the most proximal external support provided to adolescents. Parental emotional support involves expressing love, concern, and care for their children. Adolescents with higher levels of parental emotional support are more likely to develop secure attachments, thereby enhancing their level of cognition and mental health (36). Positive parental emotional expression can promote positive interactions between parents and children (37, 38). Good parent-child interactions contribute to the development of children’s emotional regulation abilities. Morris et al. further emphasized that the higher the level of parental emotional support, the better adolescents’ emotional regulation abilities (12).

Emotional regulation illuminates adolescent mental health outcomes (9). Adolescents with proficient emotional regulation skills are better equipped to cope with negative emotions and daily challenges, enhancing their cognition function and ability to bounce back from setbacks. Conversely, adolescents who lack effective emotional regulation abilities are more susceptible to the detrimental effects of stress and setbacks, increasing their susceptibility to mental health problems (39). Thus, a strong correlation exists between parental emotional support and mental health issues (40, 41). In sum, parental emotional neglect may expose children to considerable adversity (42), potentially leading to mental health issues in such children (13).

However, not all individuals who face adversity will develop mental health problems, indicating the presence of protective factors. Research conducted in Western countries has suggested that adolescents with well-functioning psychological cognition can maintain good mental health despite a lack of parental emotional support (18). Well-functioning psychological cognition may play a positive role in offsetting external deficiencies (43). According to social cognitive theory (44), the path linking parental emotional support with adolescent mental health usually passes through psychological cognition. Functioning as the most direct form of social support, parental emotional support catalyzes the psychological processes that exert an influence on individual-level behavior and performance. In this study, we sought an individual-level concept that not only reflects the personal processes shaped by the environment and social dynamics but also embodies the traits of psychological cognition.

One of the best potential candidates for such a concept is “self-efficacy” (45), referring to the subjective perception of an individual’s abilities in specific tasks or goals. It serves as a robust candidate for a mediator of the relationship between environmental and social factors and an individual’s mental health, owing the distinctive position of self-efficacy in theory and research (14). Self-efficacy is conceived as a mediator by virtue of its connection of an individual’s experiences and perceptions to actual performance (46), a role extensively validated in numerous studies (47–49). However, this role is yet to be confirmed among Hong Kong adolescents (50). Therefore, this study examines the potential mediating role of self-efficacy in the pathway between parental emotional support and adolescents’ mental health outcomes, including both psychological and somatic symptoms, within the culturally hybridized milieu of Hong Kong.

### 2.3 Moderating role of SES

Although a lack of parental emotional support may cause mental health problems, it does not affect all adolescents equally. SES serves as an important external material safeguard during adolescent development. Research indicates that individuals who grow up in a lower SES family are more likely to experience mental health issues (8), primarily due to their heightened exposure to adversity, which makes them more prone to depression or anxiety (51, 52). However, some studies suggest that adolescents from higher SES backgrounds face a heightened risk of mental health problems due to the stigma associated with seeking help (19). These findings are inconsistent.

SES typically indicates the possession of social and economic resources. Higher SES is typically associated with greater access to resources and opportunities, providing adolescents with increased support and protection and contributing to developing their self-efficacy (53, 54). These resources may include superior education, healthcare, and social support. Despite the shame experienced by high-SES adolescents when seeking help for mental health, they nevertheless have more access to these resources than do people from low-SES background. Further, adolescents from higher SES backgrounds may be more likely to receive emotional support from their parents, thus enhancing their level of self-efficacy. In contrast, lower SES may limit parents’ capacity to provide support, leading to a lack of appropriate guidance and support for adolescents in coping with stress and adversity and reducing their level of self-efficacy (18). Therefore, we propose that SES may moderate both the first link in the mediation process and the direct link between parental emotional support and mental health problems.

### 2.4 Mental health problems among adolescents in Hong Kong

The mental health issues among Hong Kong’s youth have escalated, becoming an increasingly severe societal concern. Recent study has revealed a concerning prevalence of adolescent mental health issues in Hong Kong, with nearly half of adolescents (47.3%) reporting at least one episode of suicidal ideation (55). This highlight**s** the considerable mental health challenges experienced by youth in Hong Kong. These alarming issues are likely tied to the unique context of Hong Kong. Hong Kong is characterized by its a highly competitive society and the fusion of Eastern and Western cultures (56). The high-pressure environment, marked by high population density and the highest living costs globally (57), imposes significant psychological stress on its people, including a financial burdens, housing difficulties, and so on. Moreover, as a society that values Confucian principles and embodies a blend of Eastern and Western cultural influences, Hong Kong places exceptionally high expectations on children’s academic achievements. These overwhelming social pressures and expectations not only undermine family quality of life and well-being but also further impair the mental health of adolescents (58).

Shek and Siu have also noticed that the developmental environment for adolescents in Hong Kong is permeated with a sense of unhappiness (59). This is manifested in an excessive emphasis on academic achievement at the expense of overall life satisfaction, coupled with issues such as poverty, problematic parenting, and a decline in family well-being. These issues have profound impacts on adolescents’ academic performance, social interactions, and overall quality of life, potentially leading to long-term psychological problems and social dysfunction (56). Previous research has shown that stronger familial bonds are associated with reduced levels of depression and anxiety among adolescents in Hong Kong (58). Under the perceptive of ecosystem, our study attempts to explore the interplay between parental emotional support, adolescent self-efficacy and socioeconomic status in influencing mental health among Hong Kong’ youth.

### 2.5 The present study

**Figure 1:**
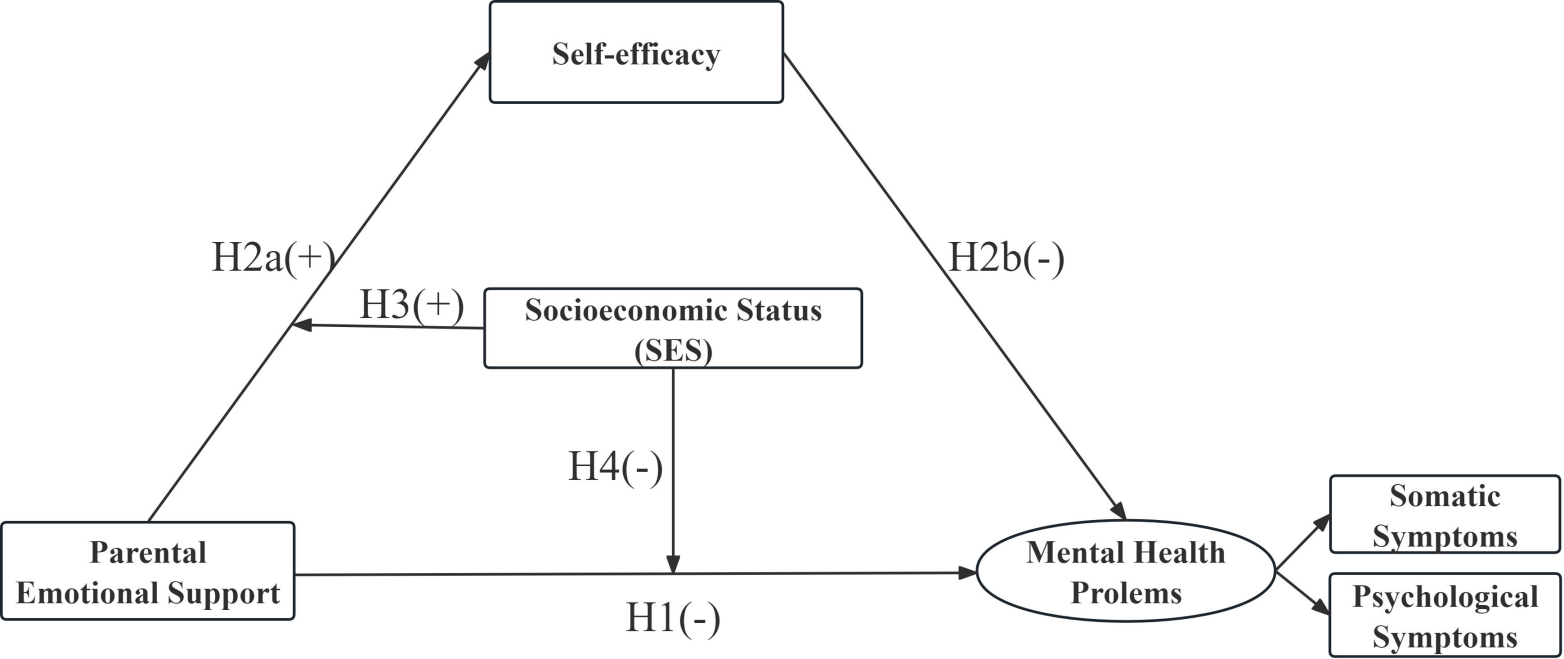
Hypothesized model.

To bridge the gaps in the literature, this study investigated the link between parental emotional support and the two dimensions of mental health problems among adolescents in Hong Kong, focusing on the mediating role of self-efficacy and the potential moderating effects of SES (**Figure 1**). Specifically, the study addressed the following questions and generated four hypotheses (H1 to H4):

1. What is the relationship between parental emotional support and mental health problems?
2. H1: Parental emotional support negatively affects adolescent mental health problems.
3. Does self-efficacy play a mediating role in this relationship?
4. H2: Parental emotional support has a positive impact on self-efficacy (H2a), and self-efficacy negatively affects mental health problems (H2b).
5. Does SES moderate the relationship between parental emotional support and mental health problems via self-efficacy?
6. H3 and H4: SES moderates the positive association between parental emotional support and self-efficacy (H3) and the negative association between parental emotional support and mental health problems (H4).

This study contributes to advancing theoretical knowledge in the field of multicultural education, as well as adding to the growing body of research on the relationship between parental emotional support, self-efficacy, and adolescent mental health in the context of Hong Kong. Additionally, this study constitutes a substantial reference source for addressing adolescents’ mental health problems in Hong Kong, with implications for educational and public health policies.

## 3 Methods

### 3.1 Participants

The Program for International Student Assessment (PISA), administered by the Organization for Economic Cooperation and Development (OECD), is a triennial international study. It primarily evaluates the academic performance (e.g., reading, mathematics, and science) and overall well-being of 15-year-old students. The program aims to promote high-quality and equitable learning outcomes, aligning with one of the Sustainable Development Goals for Education (60). PISA data are available in SAS and SPSS formats that can be directly downloaded from the PISA website (61), allowing researchers to access anonymized data for academic purposes. This study utilized the data from Hong Kong in the seventh cycle of PISA 2018.

The sampling procedure in PISA involved a two-stage stratified sampling design to ensure a representative sample of the target population (60). In the first stage, schools were selected with probability proportional to size, meaning that larger schools had a higher chance of being selected. In the second stage, a random sample of 15-year-old students within each selected school was chosen to participate in the assessment. Hence, a total of 6,037 students were collected from 152 secondary schools in Hong Kong. After data cleaning, our sample extracted the information of 3,705 students from 109 schools in Hong Kong. The Hong Kong sample was designed to be representative of the 15-year-old student population in the region, accounting for various types of schools (public and private government-dependent) and different socioeconomic backgrounds. The use of this stratified sampling approach ensures that the findings from our analysis can be generalized to the broader adolescent population in Hong Kong.

### 3.2 Measures

This section describes the variables from PISA 2018 used in this study, which were selected from the student and well-being questionnaires.

#### 3.2.1 Parental emotional support

Three items were used to measure parental emotional support from the student questionnaire (“My parents support my educational efforts and achievements,” “My parents support me when I am facing difficulties at school,” and “My parents encourage me to be confident”). Participants were required to respond on a four-point scale ranging from 1 = strongly disagree to 4 = strongly agree, with higher scores indicating greater levels of parental emotional support (62, 63). The internal consistency of the parental emotional support scale was 0.758.

#### 3.2.2 Self-efficacy

Five items were used to measure participants’ self-efficacy from the student questionnaire (“I usually manage one way or another,” “I feel proud that I have accomplished things,” “I feel that I can handle many things at a time,” “My belief in myself gets me through hard times,” and “When I’m in a difficult situation, I can usually find my way out of it”). Participants were required to respond on a four-point scale ranging from 1 = strongly disagree to 4 = strongly agree, with higher scores indicating greater levels of self-efficacy (64). The internal consistency of the self-efficacy scale was 0.812.

#### 3.2.3 Mental health problems

The PISA adopted the Health Behavior in School-Aged Children (HBSC) scale to measure the non-clinical mental health problems of students, including two dimensions that encompass psychological and somatic symptoms (65). Nine items from the well-being questionnaire were used to measure students’ mental health problems, including psychological symptoms (“feeling depressed,” “irritability or bad temper,” “feeling nervous,” “difficulties in getting to sleep,” and “feeling anxious”) and somatic symptoms (“headache,” “back pain,” “stomach pain,” and “feeling dizzy”). Participants were required to respond on a five-point scale ranging from 1 = rarely or never to 5 = about every day, with higher scores indicating a less positive statement of mental health outcomes. The internal consistency of the mental health problems scale indicated acceptable reliability (Cronbach’s alpha = 0.792 for psychological symptoms and 0.827 for somatic symptoms).

#### 3.2.4 Moderator

SES was defined as economic, social, and cultural status (ESCS) in PISA and was derived from three variables: “parents’ highest level of education,” “parents’ highest occupational status,” and “home possessions.” The index average was zero, and the standard deviation was one across OECD countries. Higher scores indicate better SES (60).

#### 3.2.5 Covariates and missing data

Except for the core variables, we also included several covariates as they related to mental health problems. We included one student-level covariate: student gender. Student gender was scored as 1 = female and 2 = male. Owing to the nested nature of the data, we also used three school-level covariates: school type, location, and size (66, 67). Student and school sampling weights were considered for descriptive statistics and model estimation.

The Hong Kong dataset showed a proportion of missing data of small (3.36%) to large (22.4%) for several variables. We deleted the observations with any missing information in the variable of mental health problems, which reduced the sample size from 6,037 to 3,705 when conducting the final analytical procedure (i.e., the moderated mediation models). To address the issue of missing data in other variables, we employed multiple imputation by chained equations (MICE), generating 20 imputed datasets. The dataset was addressed using full information maximum likelihood, as recommended for PISA-related research (68). This approach involves estimating the variances of all variables in the model without imputing missing values, using all available data for each parameter estimation. Studies have shown that this method outperforms listwise/pairwise deletion and simple imputation, yields robust standard error estimates (69), and is resilient to non-normality (70).

#### 3.2.6 Analytical procedure

First, we reported the descriptive analyses and Pearson correlations between SES, parental emotional support, self-efficacy, and student mental health problems. Hierarchical linear modeling was applied owing to the nested nature of the 2018 PISA dataset, with students nested within schools (71). We used a two-level hierarchical linear model, with students at the lower level and schools at the higher level. We performed an unconstrained or null model, without any student- and school-level variables, to decompose the variance in the outcome variable between and within the clusters (e.g., schools). This decomposition of variance helps to determine the proportion of variance at each level, known as the intraclass correlation coefficient (ICC). An ICC value greater than 0.01 indicates the appropriateness of aggregating the student-level data to the school level (72).

Using simple mediation analysis, the mediation models were then tested to determine whether the relationship between parental emotional support and mental health problems could be explained by the influence of parental emotional support on student self-efficacy and that of self-efficacy on mental health problems. Finally, we utilized conditional process analysis to examine the moderated mediation models. This analytical approach integrates both moderation and mediation within a unified theoretical framework and assesses the indirect effects under different levels of the moderator variable (73). Bootstrapping analysis with 5,000 replicates was conducted to verify the significance of the (moderated) mediation paths.

Data cleaning and all of the aforementioned analytical procedures were conducted using R version 4.0.3 (74). Specifically, ICC was tested via a psychometric package, moderation and mediation modeling was conducted via the glmmTMB package, and weighting values were obtained by multiplying the student-level weights by the corresponding school-level weights, as the glmmTMB function does not permit manipulating school- and student-level weights separately.

## 4 Results

### 4.1 Descriptive statistics

**Table 1** presents the descriptive statistics for the 3,705 students in 109 schools. The ICC was 2.02% for psychological symptoms and 1.26% for somatic symptoms, indicating that 2.02% of the total variance in psychological symptoms was attributable to schools and 1.26% of the total variance in somatic symptoms was attributable to schools.

**Table 1:** Descriptive statistics of 3,705 students at 109 schools.

Variables	Mean (SD)/N(%)
Gender	
Male	1,913 (51.6%)
Female	1,792 (48.4%)
Psychological symptoms	2.266 (1.051)
Somatic symptoms	1.966 (0.893)
SES	–0.557 (1.028)
Parental emotional support	3.097 (0.628)
Self-efficacy	2.830 (0.490)
School size	763.147 (216.651)
School location	
A village, hamlet, or rural area (fewer than 3,000 people)	2 (1.8%)
A small town (3000 to c. 15,000 people)	2 (1.8%)
A town (15 000 to c. 100,000 people)	17 (15.6%)
A city (100, 000 to c. 1,000,000 people)	41 (37.6%)
A large city (with over 1,000,000 people)	47 (43.1%)
School type	
Public	12 (11.0%)
Private government-dependent	97 (89.0%)

### 4.2 Testing for mediation effects

The mediation analysis results, presented in **Table 2**, assess the mediating role of student self-efficacy in the relationship between parental emotional support and the two dimensions of student mental health problems—somatic symptoms and psychological symptoms. Model 1 shows that parental emotional support significantly and negatively predicted somatic symptoms (b = -0.545, SE = 0.024, p < 0.001), indicating that higher levels of parental emotional support are associated with fewer somatic symptoms in adolescents. Similarly, Model 2 demonstrates that parental emotional support also significantly and negatively predicted psychological symptoms (b = -0.678, SE = 0.012, p < 0.001), showing a reduction in psychological symptoms with increased parental support.

**Table 2:** Testing the mediation effect of student self-efficacy on mental health problems.

	Model 1 (Somatic symptoms)	Model 2 (Psychological symptoms)	Model 3 (Self-efficacy)	Model 4 (Somatic symptoms)	Model 5 (Psychological symptoms)
Parental emotional support	-0.545*** (0.024)	-0.678*** (0.012)	0.562*** (0.021)	-0.281 *** (0.029)	-0.389*** (0.031)
SES	-0.072*** (0.020)	-0.077*** (0.019)	0.088*** (0.014)	-0.036*** (0.017)	-0.067*** (0.018)
Self-efficacy				-0.291*** (0.024)	-0.295*** (0.027)
R-square	0.178	0.171	0.194	0.208	0.222
F	365.609***	347.424***	408.569***	333.310***	360.297***

Each model adjusted for covariates (i.e., gender, school size, school location, and school type); ***p < 0.001.

In Model 3, the analysis shows a significant positive association between parental emotional support and student self-efficacy (b = 0.562, SE = 0.021, p < 0.001), suggesting that students who perceive higher parental emotional support tend to report higher levels of self-efficacy.

Model 4 examines the effects of both parental emotional support and self-efficacy on somatic symptoms. The results indicate that both parental emotional support (b = -0.281, SE = 0.029, p < 0.001) and self-efficacy (b = -0.291, SE = 0.024, p < 0.001) are significant predictors, with higher levels of each associated with fewer somatic symptoms. This suggests that self-efficacy partially mediates the relationship between parental emotional support and somatic symptoms.

Model 5 presents similar findings for psychological symptoms, where both parental emotional support (b = -0.389, SE = 0.031, p < 0.001) and self-efficacy (b = -0.295, SE = 0.027, p < 0.001) were negatively associated with psychological symptoms. This confirms the mediation effect of self-efficacy in the relationship between parental emotional support and psychological symptoms, indicating that part of the effect of parental emotional support on psychological symptoms operates through its influence on self-efficacy.

### 4.3 Testing for moderated mediating effects

The moderated mediation analysis, as detailed in **Table 3**, explores whether SES alters the strength of these mediation effects. The results demonstrate that SES indeed moderates the relationship between parental emotional support and self-efficacy. Specifically, Model 6 shows that the interaction between parental emotional support and SES is significantly positive (b = 0.006, p < 0.001), indicating that the positive effect of parental emotional support on self-efficacy is more pronounced in adolescents with higher SES. This suggests that SES amplifies the mediation effect, making the pathway from parental emotional support to improved mental health outcomes through self-efficacy stronger for adolescents from higher SES backgrounds. However, the analysis also shows that SES does not significantly moderate the direct relationship between parental emotional support and somatic symptoms, as indicated by the non-significant interaction term in Model 7 (b = -0.019, p = 0.127). In contrast, for psychological symptoms, Model 8 reveals a significant negative interaction (b = -0.033, p < 0.001), suggesting that the direct negative effect of parental emotional support on psychological symptoms weakens as SES increases.

**Table 3:** Testing the moderated mediation effects of SES.

	Model 6 (Self-efficacy)	Model 7 (Somatic symptoms)	Model 8 (Psychological symptoms)
Parental emotional support	0.566*** (0.033)	-0.157 *** (0.035)	-0.302*** (0.037)
SES	0.068*** (0.014)	-0.112*** (0.015)	-0.139*** (0.017)
Self-efficacy		-0.297*** (0.036)	-0.293*** (0.027)
Parent emotional support × SES	0.006*** (0.002)	-0.019 (0.014)	-0.033*** (0.012)
R-square	0.202	0.209	0.224
F	340.157***	267.914***	288.124***

Each model adjusted for covariates (i.e., gender, school size, school location, and school type); ***p < 0.001.

To further elucidate these relationships, **Table 4** presents the conditional effects of parental emotional support on mental health outcomes across different levels of SES. The analysis reveals that the negative relationship between parental emotional support and psychological symptoms is significant at all SES levels, but the strength of this relationship increases with higher SES. For somatic symptoms, however, the direct negative effect of parental emotional support becomes significant only at higher SES levels, indicating that the protective effect of parental support against somatic symptoms is more evident in adolescents from higher SES backgrounds. Additionally, the indirect effects of parental emotional support on both somatic and psychological symptoms via self-efficacy are significant across all SES levels, with the effect size slightly increasing with SES. This suggests that the combined effect of parental emotional support and self-efficacy in reducing mental health symptoms is stronger for adolescents from higher SES backgrounds, highlighting the nuanced role that SES plays in moderating both direct and indirect pathways in this context.

**Table 4:** Conditional effects of parental emotional support on mental health problems.

	Somatic symptoms		Psychological symptoms	
	Coefficient	[LB, UB]	Coefficient	[LB, UB]
conditional direct effect				
Mean - 1 × SD on SES	-0.117	[-0.267, 0.033]	-0.250	[-0.382, -0.118]
Mean on SES	-0.143	[-0.293, 0.007]	-0.284	[-0.416, -0.152]
M + 1 × SD on SES	-0.169	[-0.319, -0.019]	-0.318	[-0.450, -0.186]
conditional indirect effect				
M - 1 × SD on SES	-0.165	[-0.325, -0.005]	-0.163	[-0.295, -0.031]
Mean on SES	-0.167	[-0.327, -0.007]	-0.165	[-0.297, -0.033]
M + 1 × SD on SES	-0.169	[-0.329, -0.009]	-0.167	[-0.299, -0.035]

**Figure 2:**
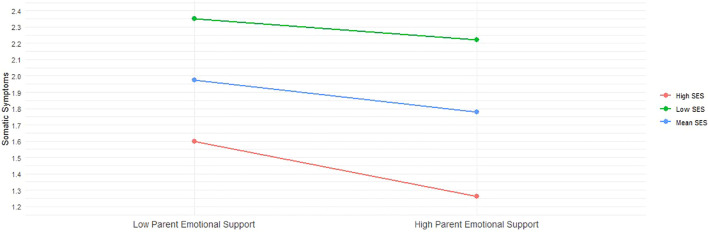
Interaction between Parental Emotional Support and Somatic Symptoms across SES Levels.

**Figure 3:**
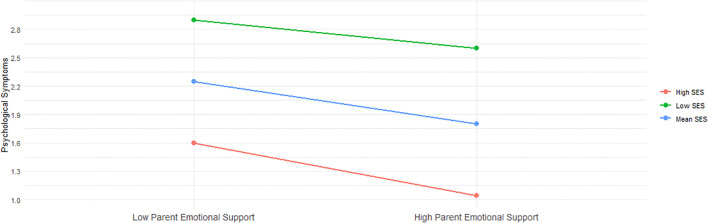
Interaction between Parental Emotional Support and Psychological Symptoms across SES Levels.

In addition to these results, we plotted the predicted somatic and psychological symptoms against parental emotional support, stratified by socioeconomic status (SES). Specifically, we examined these relationships for individuals with low SES (one standard deviation below the mean), mean SES, and high SES (one standard deviation above the mean). **Figure 2** reveals that while somatic symptoms remain relatively stable among adolescents with low and mean SES, regardless of the level of parental emotional support, there is a significant reduction in these symptoms among high SES adolescents as parental support increases. This suggests that the protective benefits of parental emotional support against somatic symptoms are predominantly evident in the high SES group. Conversely, **Figure 3** demonstrates a consistent negative association between increased parental emotional support and psychological symptoms across all three SES groups. This relationship is statistically significant for both groups, indicating that parental emotional support serves as a crucial buffer against psychological symptoms irrespective of the adolescent’s socioeconomic background. These two figures underscore the differential impact of socioeconomic factors on the efficacy of parental emotional support in mitigating mental health issues among adolescents.

## 5 Discussion

In this study, we developed a model to examine the relationship between parental emotional support and mental health problems among adolescents in Hong Kong and to explore the potential mediating role of self-efficacy and the moderating effect of SES in this relationship. Our findings showed that parental emotional support had a significantly negative direct effect on the two dimensions of adolescent mental health problems (i.e., psychological and somatic), supporting H1. When considering the mediating impact of self-efficacy, we found a significant indirect effect of parental emotional support on psychological and somatic symptoms, thus supporting H2. Finally, our results showed significant moderating effects of SES on the pathway from parental emotional support to self-efficacy, as well as on the pathway from parental emotional support to psychological symptoms, partially supporting H3 and H4. Our findings contribute to the literature on parental emotional support and adolescent mental health. While much research has focused on adolescent psychological symptoms, our study reveals that self-efficacy mediates the impact of parental emotional support on both two dimensions of mental health problems. Additionally, SES does not moderate somatic symptoms, highlighting important contextual and developmental differences.

### 5.1 Parental emotional support and mental health problems

Our study enhances the understanding of adolescent mental health by incorporating an often-overlooked facet: somatic symptoms, alongside the more commonly examined psychological symptoms. Inconsistent with earlier studies that primarily focused on somatic symptoms as early indicators of mental health (28–30), our findings suggest that in the Hong Kong context, psychological symptoms are not only prevalent but also more discernible and reportable among adolescents. This indicates that a comprehensive evaluation of adolescent mental health should place greater emphasis on psychological symptoms, as they more accurately reflect the overall mental well-being of adolescents in this specific societal setting.

Further, aligning with research (40, 41, 75), our study demonstrated a negative association between parental emotional support and the two dimensions of mental health problems in Hong Kong. According to attachment theory (35), emotional attachment to parents is essential for establishing good psychological functioning throughout development (76), aiding adolescents in enhancing emotion regulation. Adolescents with proficient emotion regulation skills are better equipped to cope with negative emotions and daily challenges, thereby enhancing their self-efficacy and ability to bounce back from setbacks. Furthermore, in this study, 15-year-old adolescents may have been facing a major school-related transition, which might have increased their risk of mental health problems (77).

### 5.2 Mediation role of self-efficacy

In the framework of social cognition theory, the results demonstrated that self-efficacy, as the potential mediating candidate, can account for the impact of parental emotional support on mental health problems, highlighting its significance as a critical factor in promoting adolescent mental health. Specifically, parental emotional support not only directly influences the mental health problems of adolescents but also exerts more profound effects by cultivating self-efficacy (13).

Self-efficacy, functioning as the core belief system regarding an individual’s capabilities, directly shapes the attitudes and behaviors of adolescents when confronting challenges. Individuals with elevated self-efficacy are more prone to exhibiting heightened confidence and positivity, courageously facing challenges rather than resorting to negative avoidance (15). For instance, during the COVID-19 pandemic, a survey by Cattelino et al. found that adolescents with higher self-efficacy tend to employ more coping strategies when dealing with negative emotions, thereby enhancing their sense of well-being (47).

Considering the context of Hong Kong, research found that during childhood, self-efficacy fails to significantly mediate the relationship between parental emotional support and psychological symptoms (50). Nevertheless, our study observed a significant mediating effect of self-efficacy between parental emotional support and both the two dimensions of mental health (i.e., psychological and somatic symptoms) during adolescence. A plausible reason for this discrepancy is that adolescents at the age of 15 are undergoing a critical phase marked by a sharp increase in self-development, during which self-efficacy gains prominence (77). Parental emotional support may be more effective in improving adolescents’ psychological and somatic symptoms by enhancing their self-efficacy. Additionally, Hong Kong’s mixed cultural context may shape parenting style, blending traditional Chinese involvement with Western encouragement of independence, which bolsters adolescent self-efficacy.

### 5.3 Moderating role of SES

This study found that SES plays a moderating role in the first part of the mediation process (parental emotional support → self-efficacy). Lower parental emotional support is more strongly associated with lower self-efficacy in adolescents with low SES, leading to both dimensions of mental health problems. As SES increases, these differences tend to gradually intensify. These findings indicate that adolescents from disadvantaged backgrounds may experience an amplified negative impact of risk factors on their mental health (19). This is consistent with neuroscientific research (78), which demonstrated that individuals from lower SES backgrounds might face challenges owing to limited resources, potentially resulting in delayed neurodevelopment and negative implications for self-efficacy cultivation.

Additionally, the findings of this study revealed that SES plays a moderating role in the direct path and the first stage of the mediation model (parental emotional support → self-efficacy → mental health problems). Specifically, low SES amplifies the negative association between parental emotional support and mental health problems, implying that adolescents with low SES may face greater difficulties and risks when facing mental health problems. It suggests that economic differences contribute to mechanisms underlying adolescent psychological symptoms. Moreover, SES was identified as a significant factor in the inequality of adolescent mental health, which aligns with previous studies (79–81). Higher SES is often associated with greater access to resources and opportunities, providing adolescents with more emotional support and resources, helping them cope with negative emotions, and reducing the risk of behavioral problems (82).

However, SES was not found to moderate the relationship between parental emotional support and somatic symptoms, which contradicts previous studies (83). There are two possible reasons for this discrepancy. First, financially supported by the Hong Kong government, adolescents from disadvantaged socioeconomic backgrounds may face greater barriers to accessing healthcare and health resources than those from advantaged socioeconomic backgrounds (84). To diminish the inequality, the Community Care Fund, initiated by the Hong Kong government (85), has been providing financial assistance to economically disadvantaged students. This financial aid primarily covers essential expenses, such as basic educational and medical expenses. Therefore, it is more likely to mitigate the adverse effects of low SES on the relationship between parental emotional support and the somatic symptoms of adolescents rather than the psychological symptoms, according to Maslow’s hierarchy of needs (86). Another possible explanation for this inconsistency, based on the risk accumulation model (87), is that the risk of somatic symptoms may begin at the prenatal stage and accumulate throughout adulthood, ultimately affecting the physical health of individuals. As the participants were still young (i.e., 15 years of age), the moderating effect of SES on somatic symptoms may have not yet significantly manifested.

### 5.4 Limitations and future research

Our study is subject to some limitations. First, the data were obtained from self-report measures from the participants. It is generally recognized that such results may be influenced by various factors, such as different perspectives between parents and adolescents, as highlighted by Nunes et al. (88). To address this limitation, future studies could consider including measures of parental reported emotional support and exploring specific divisions of labor between parents.

Second, the data collected in our study were cross-sectional, which limits establishing causal relationships. Future research should incorporate longitudinal designs with multiple time points to provide more comprehensive understanding of the potential effects of parental emotional support on adolescent mental health; doing so would allow for the examination of temporal associations and the exploration of developmental trajectories.

Finally, the data for this study were collected before the COVID-19 pandemic. Since the onset of the pandemic, mental health issues among adolescents have markedly increased (89). The social isolation experienced during this time may have led adolescents to rely more heavily on emotional support from their parents, further validating our model. Future research should replicate this study in the post-pandemic context to verify and compare the results. This would help determine whether the observed relationships hold true across different contexts and periods.

### 5.5 Implications for practice

Despite some limitations of this study, the findings extend understanding of the impact of parental emotional support on adolescent mental health problems in the context of Hong Kong and have some practical implications for addressing mental health issues beyond Hong Kong youth.

First, the findings of this study reveal the protective role of self-efficacy in the development and occurrence of mental health problems. Interventions targeting self-efficacy in adolescents could be a key factor because they equip them with the skills and mindset necessary to face and overcome challenges. Mindfulness-based interventions have effectively enhanced self-efficacy (90). Such interventions have several advantages, including their flexibility in accommodating different time and space constraints (91). They can also be quickly disseminated and promoted, especially in the digital era, within which various online mindfulness programs and resources exist.

Second, our study emphasizes the partial moderating role of SES in the relationship between parental emotional support, self-efficacy, and mental health problems among adolescents. These findings have significant implications for interventions and support systems targeting adolescents from low SES backgrounds. It is essential to prioritize increased attention and support for this vulnerable group as they are more likely to encounter significant challenges and risks regarding addressing mental health issues (79, 92). In recognizing the influence of economic disparities on psychological symptoms in adolescents, interventions could focus on bolstering self-efficacy and providing resources and opportunities to mitigate the adverse effects of low SES (52).

Third, our findings are of utility for regions beyond Hong Kong (18, 50, 93). These outcomes imply that the applicability of attachment theory and social cognitive theory extends across diverse cultural contexts (94, 95), offering valuable insights for addressing mental health concerns among adolescents in culturally complex countries. Expanding upon these findings, the World Health Organization has the potential to strengthen its focus on economically disadvantaged areas, particularly those necessitating heightened support for adolescents (96). By emphasizing the significance of parent-child relationships and fostering adolescent self-efficacy, the mental health of adolescents can be enhanced, thereby presenting promising pathways toward the achievement of health equity.

## 6 Conclusions

This study examines the association between parental emotional support, self-efficacy, and mental health problems among adolescents in Hong Kong, finding relationships similar to those observed in studies conducted in Western countries (97). The findings highlight that parental emotional support negatively impacts adolescent mental health problems primarily through its influence on self-efficacy. Additionally, SES has a moderating role in the relationships between parental emotional support and self-efficacy, as well as between parental emotional support and psychological symptoms. For adolescents from disadvantaged backgrounds, parental emotional support is a relatively strong predictor of mental health problems. The study also illuminates the internal mechanisms by which parental emotional support influences adolescent mental health problems in Hong Kong, and it underscores the importance of targeted interventions by educators and policymakers to provide precise support for disadvantaged adolescents, to enhance their self-efficacy (98).

## Data Availability

Publicly available datasets were analyzed in this study. This data can be found here: https://www.oecd.org/pisa/data/2018database/.

## Footnotes

1 Notably, to enable comprehensive understanding of students’ overall health, PISA collected indicators covering both objective and subjective aspects. In our study, we focused on two dimensions of subjective mental health indicators.
